# In Vitro Time Efficiency, Fit, and Wear of Conventionally- versus Digitally-Fabricated Occlusal Splints

**DOI:** 10.3390/ma15031085

**Published:** 2022-01-30

**Authors:** Sebastian Berthold Maximilian Patzelt, Marei Krügel, Christian Wesemann, Stefano Pieralli, Julian Nold, Benedikt Christopher Spies, Kirstin Vach, Ralf-Joachim Kohal

**Affiliations:** 1 Private Dental Clinic, Am Dorfplatz 3, 78658 Zimmern ob Rottweil, Germany; 2 Medical Center—University of Freiburg, Center for Dental Medicine, Department of Prosthetic Dentistry, Faculty of Medicine, University of Freiburg, Hugstetter Str. 55, 79106 Freiburg, Germany; marei.kruegel@uniklinik-freiburg.de (M.K.); christian.wesemann@uniklinik-freiburg.de (C.W.); stefano.pieralli@uniklinik-freiburg.de (S.P.); julian.nold@uniklinik-freiburg.de (J.N.); benedikt.spies@uniklinik-freiburg.de (B.C.S.); ralf.kohal@uniklinik-freiburg.de (R.-J.K.); 3 Medical Center—University of Freiburg, Institute of Medical Biometry and Statistics, Faculty of Medicine, University of Freiburg, Stefan-Meier-Str. 26, 79104 Freiburg, Germany; kv@imbi.uni-freiburg.de

**Keywords:** occlusal splints, CAD/CAM, manufacturing process, digital workflow, wear, fit, time efficiency

## Abstract

The purpose of the study was to compare conventional to digital workflows of occlusal splint production regarding time efficiency, overall fit, and wear. Fifteen Michigan splints were fabricated with a conventional and digital method. The duration for the dentist’s and the dental technician’s workload was recorded. Subsequently, the overall fit was examined with a four-level score (1–4). Paired t-tests were used to compare the time results for the conventional and digital workflows and the sign test to compare the overall fit. The mean time (16 min 58 s) for computerized optical impressions was longer than for conventional impressions (6 min 59 s; *p* = 0.0001). However, the dental technician needed significantly less mean time for the digital splint production (47 min 52 s) than for the conventional (163 min 32 s; *p* = 0.001). The overall fit of the digitally-fabricated splints was significantly better compared to the conventionally-fabricated splints (*p* = 0.002). There was no impact of the different materials used in the conventional and digital workflow on the wear (*p* = 0.26). The results suggest that the digital workflow for the production of occlusal splints is more time efficient and leads to a better fit than the conventional workflow.

## 1. Introduction

Craniomandibular dysfunctions (CMD) have long been recognized as the leading cause of non-dentogenic pain in the orofacial region with a prevalence of 20–50% among the adult population [1,2]. The multifactorial etiology of CMD requires an interdisciplinary treatment between dentists, physiotherapists, psychologists, and orthopedic specialists [3]. The recommended initial dental intervention is a reversible treatment with occlusal splints relieving tensions and pain in 50–80% of the patients [1,4,5,6,7,8,9]. Centric splints with individualized occlusal surfaces, also termed Michigan splints, are considered the gold standard regarding their risk-benefit ratio [6,10,11]. Creating an ideal occlusion through a splint therapy with resulting harmonization and relaxation of the masticatory system leads to a reorganization of intramuscular and intraarticular functional patterns and pain relief of the strained muscle groups [12,13,14].

Michigan splints are made of an acrylic resin base plate with an even bite plane in all supporting zones. Features of these stabilizing bite splints are the canine guidance and the “freedom in centric” occlusion concept [15,16].

The conventional workflow for the production of Michigan splints [alginate impressions, bite registration, cast fabrication, splint wax-up, and powder-liquid mixtures of polymethylmethacrylates (PMMA)] is well established. However, the digital production workflow has developed steadily in recent years [17]. The digital workflow for the fabrication of splints comprises computerized optical impressions of the upper and lower jaw, a digital bite registration, a computer-aided design (CAD), and a computer-aided manufacturing (CAM). Whereas in the early years of computerized optical impression making, a full-arch scan led to high inaccuracies, computerized optical full-arch scans have in the meanwhile reached comparable results to conventional impressions in terms of trueness and precision [18,19,20,21]. The CAM process can either be performed by subtractive techniques such as milling of PMMA blanks or additive techniques such as line-stereolithography, digital light processing, or material jetting [22].

Obviously, the digital workflow is preferred by patients as the intraoral use of a small scanner wand increases the patient comfort compared to conventional impression making [23,24,25,26]. In the case of a loss or fracture of the splint, the stored data might be used to produce a new splint without redoing the impression [17,27,28]. A success of the splint therapy, however, seems to be independent of the manufacturing process. Both splint fabrication methods are equally successful in the treatment of CMD [27,28,29].

Another aspect is the influence of different splint materials on the wear. Studies investigating the wear of different materials have come to contrary conclusions—some demonstrated higher wear of conventional splint materials others showed comparable wear of conventional and digital splint materials [30,31,32].

Finally, there is limited information available on the required time of different workflows (conventional vs. digital) to produce the splints.

Therefore, the primary aim of this investigation was to compare the duration of splint fabrication required by the dentist and dental technician using two different production workflows in a standardized in vitro setting. In addition, the overall fit and static occlusion were evaluated. The secondary aim was to evaluate the wear of different splint materials utilized over a wearing period of 1.2 million cycles in an artificial chewing simulator.

## 2. Materials and Methods

**Figure 1:**
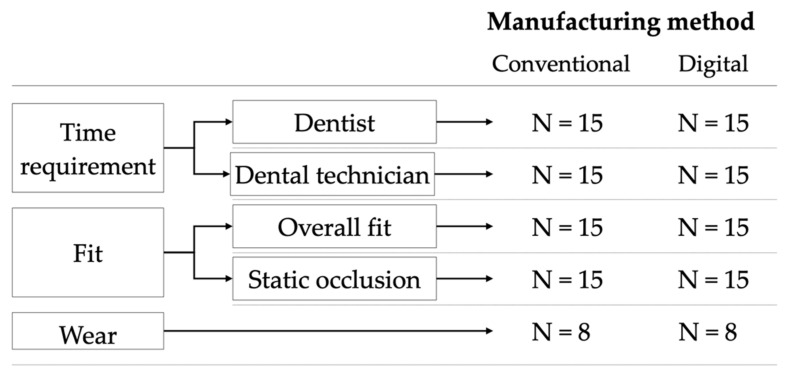
Experimental setup. N = number of Michigan splints fabricated and examined.

The present study was conducted in a dental simulation unit (P-6/3 HGB Pro V3, Frasaco, GmbH, Tettnang, Germany) with fully dentate study models of the upper and lower jaw (OK V16, UK V16, KaVo Dental GmbH, Biberach, Germany) attached to a dental chair (Estetica E 70 T, KaVo Dental GmbH, Biberach, Germany). Thirty Michigan splints were either fabricated using a conventional or a digital workflow (Figure 1).

The conventional workflow consisted of alginate impressions, a bite registration, and a conventional manufacturing process (wax-up of the splint, transfer to PMMA via injection molding) in the laboratory [33]. The digital workflow included a computerized optical impression of the upper and lower jaw, a digital bite registration (buccal scan), computer-aided designing, and computer-aided subtractive manufacturing [17]. All dental procedures of both workflows were performed by the same experienced dentist (M.K.) and all splints were manufactured by the same dental technician (S.W.).

### 2.1. Conventional Workflow

The conventional impressions of the upper and lower jaws were made with a stock tray (Pluline Rim Abdrucklöffel, Pluradent, Offenbach, Germany). The impression trays were coated according to the manufacture’s processing instructions with an alginate adhesive (Fix Tray Adhesive for Alginate Impression Material, Dentsply Sirona, Charlotte, NC, USA). The alginate material (Pluralgin Super, Pluradent) was processed according to the manufacturer’s instructions. Impressions were made and subsequently immersed in a disinfection bath (Pluraclean A, Pluradent). Next, the bite registration in the dental simulation unit was performed based on the ‘Step by Step jaw relation determination’ described by Türp [34]. The bite registration was performed with three wax plates (approximately 3-mm thickness in total; Dental Wax, Miltex, Tuttlingen, Germany), warmed in hot water and placed on the upper jaw, so that the imprints of the occlusal and incisal surfaces of the maxillary teeth were visible. A guided movement of the lower jaw led to the jaw closure. In the area of the imprints of the first molars and the canines of the lower jaw, aluminum wax (Aluwax Denture, Allendale, SC, USA) was applied to refine the imprints. After the application of the aluminum wax, the registration plate was reinserted into the dental simulation unit and the lower jaw closed until a fine imprint of the lower jaw on the aluminum wax was visible.

The alginate impressions were poured with stone (Pico-rock280, Picodent, Wipperfürth, Germany) to fabricate the master models. The stone casts of the lower jaw were mounted arbitrarily with the occlusal plane parallel to a horizontal plane in an articulator (SAM-Präzisionstechnik, Gauting, Germany). The upper jaws were fixed using the registration plate on the lower jaw. Subsequently, the Michigan splints were designed in wax, including, a flat surface with one contact point per tooth from the lower canine to the lower second molar [35]. Afterwards, the wax design was transferred into PMMA (Probase Cold, Ivoclar Vivadent, Schaan, Liechtenstein) by injection molding (a powder–liquid ratio 2:1, 4 bar, 5 min, Palajet Kulzer, Hanau, Germany). Finally, the splints were finalized and polished.

### 2.2. Digital Workflow

**Figure 2:**
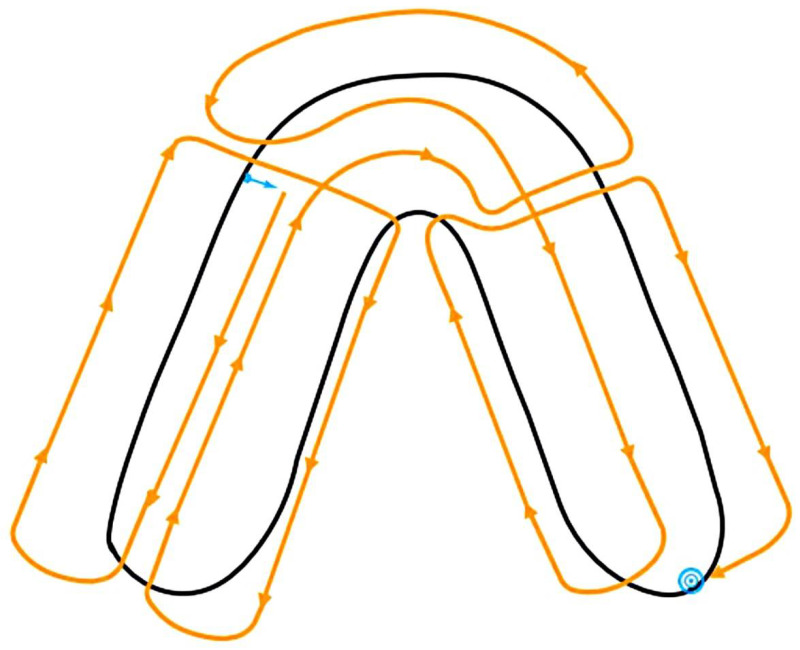
Scan path for the Cerec AC Omnicam. Blue arrow: start point, blue dot: endpoint.

The computerized optical impressions of the upper and lower jaws attached to the dental simulation unit were made with an intraoral scanner (Cerec AC Omnicam, software version 4.6.1, Dentsply Sirona, Charlotte, NC, USA) according to the scan path proposed by the manufacturer (Figure 2) [36].

The bite registration was similar to the conventional workflow. After the actual bite registration, the wax plate was trimmed on one side, so that the buccal tooth surfaces and the buccal cusps of the lower and upper jaw were visible to the scanner. A buccal bite scan was performed with these modified bite plates in situ.

The time needed for the impressions and bite registrations for the conventional and digital workflow was recorded.

After the virtual mounting of the digital models, the Michigan splint was designed in CAD software (inLab Splint design software, version 20.0.4., Dentsply Sirona) with one occlusal contact point per tooth from the lower canines to lower second molars [17]. The data of the designed splint was transferred to a milling machine (MC X5, Dentsply Sirona) and milled out of a PMMA blank (inCoris PMMA guide, Dentsply Sirona). The dental technician performed a manual finalization and polishing of the splints.

The following durations for the conventional steps of the technician were recorded:

1. Fabrication of the stone casts;
2. Mounting of the casts in the articulator;
3. Blocking out of undercuts in the model;
4. Splint design in wax;
5. Embedding the wax splint;
6. Transferring to PMMA;
7. Unbedding the PMMA splint; and
8. Occlusal adjustment, finalization and polishing;

The following durations for the digital steps of the technician were recorded:

1. Loading the scans of the upper and the lower jaws into the design software;
2. Articulating the scans with the bite scan in the design software;
3. CAD;
4. CAM;
5. Separating the splint from the blank; and
6. Finalization and polishing

### 2.3. Evaluation of the Fit

For each manufacturing method 15 Michigan splints were evaluated in terms of their overall fit and static occlusion in the dental simulation unit (Table 1). The overall fit was evaluated with a proprietary four-level scoring system. A splint with the score 1 fitted well and did not need any adaption. Score 2 indicated that a chairside subtractive adaptation including the removal of interfering interdental septa, and/or interfering splint margins and/or premature contact points was necessary. Score 3 was attributed to splints that needed additive adaption such as build-up of missing contact points and/or relining of the splints for a better retention. Score 4 indicated that the splint could not be adapted and had to be redone. The static occlusion was checked with an occlusion paper (thickness 12 µm, Hanel Okklusionsfolie, Coltene/Whaledent, Langenau, Germany). The static occlusion was also evaluated with a four-level score depending on the contact points (Table 1).

### 2.4. Evaluation of the Occlusal Wear

**Figure 3:**
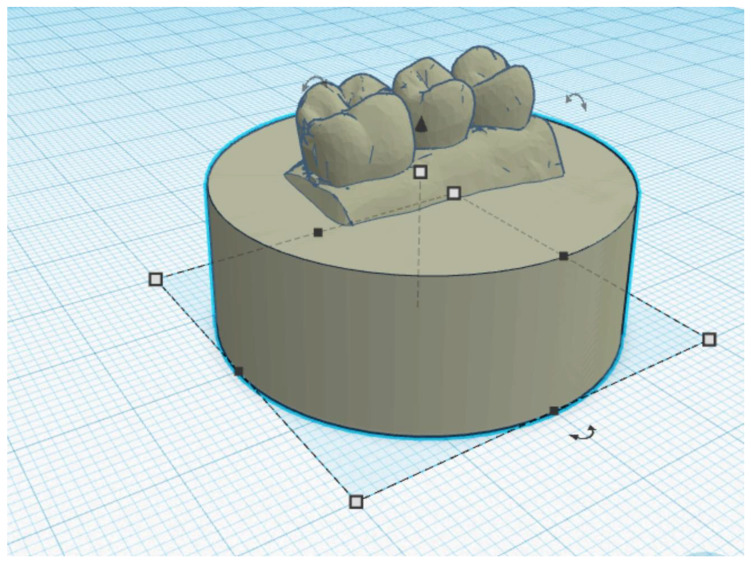
The design of the test specimen holder with teeth 14, 15, and 16 in a browser-based 3D modelling software (Tinkercad).

Eight conventionally and eight digitally-fabricated splints were used to compare the occlusal wear of the two different materials (Probase Cold and inCoris PMMA). For the occlusal wear testing, only the part of the Michigan splints from tooth region 14 to 16 (World Dental Federation notation) was applied. The intraoral scan of this region was used to fabricate test specimen holders (Grey Resin, Formlabs, Somerville, MA, USA) for the partial splints (Figure 3).

**Figure 4:**
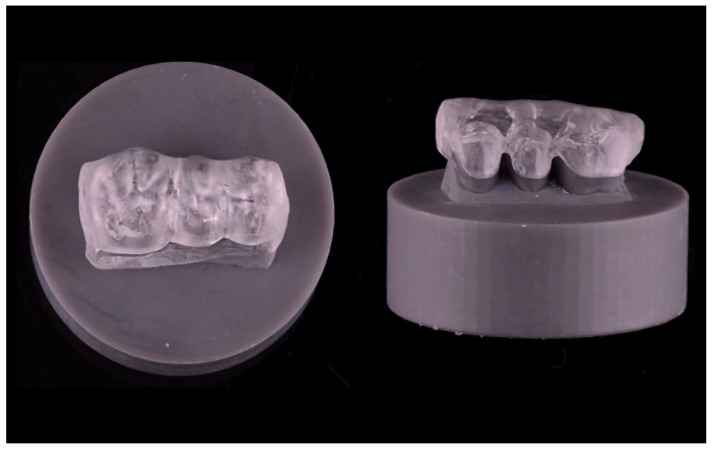
Exemplary fixed partial Michigan splint on the test specimen holder.

The holders were designed using a 3D modelling software (Tinkercad and Meshmixer, Autodesk, San Rafael, CA, USA) and then additively manufactured with a 3D printer (Form 3, Formlabs) (Figure 4). The splints were bonded to the specimen holders with a self-curing resin (Clerafil Core, Kuraray Europe, Hattersheim, Germany) for long-term retention during the artificial chewing simulation.

Before inserting the specimens in the computer-controlled chewing simulator, a surface scan of the splint area of tooth 15 was performed using a 3D optical profilometer (VR 5000, Keyence, Neu-Isenburg, Germany) and an STL data set was generated from each specimen.

**Figure 5:**
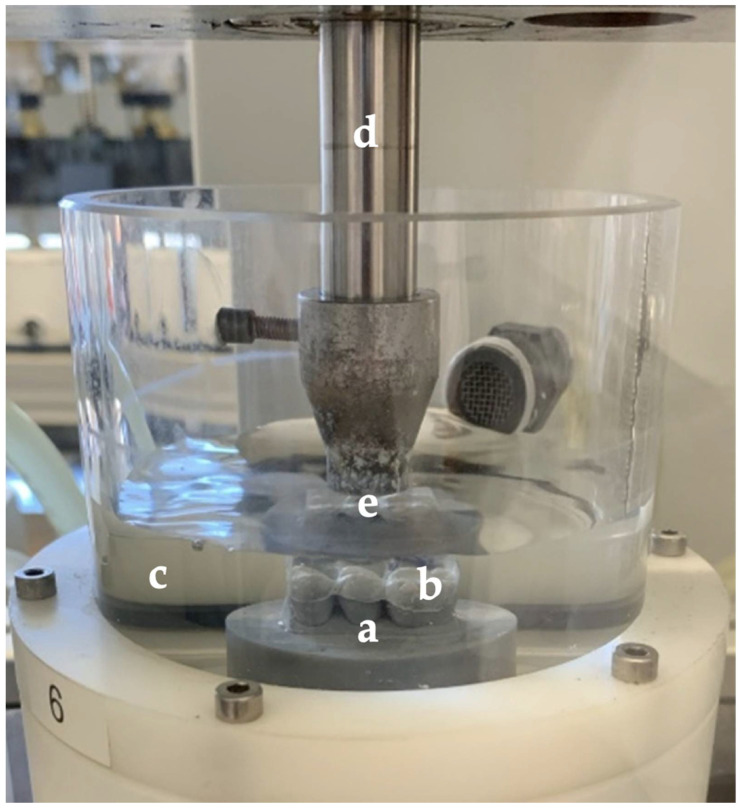
Picture of the test specimen holder (a) with the adhesively fixed partial Michigan splint (b) in the watered specimen chamber (c) of the computer-controlled chewing simulator showing the vertical bar for vertical movements (d) and the artificial antagonist (e).

A computer-controlled chewing simulator (CS-4.8, SD Mechatronik, Feldkirchen-Westerham, Germany) was used for the occlusal wear testing of the materials. The chewing simulation comprised 1.2 million chewing cycles at a frequency of 1.3 Hz (one cycle consisted of a vertical loading of the splint followed by a horizontal movement of 0.5 mm), a contact load of 50 N and thermocycling (water temperature: 5 °C/55 °C). Steatite spheres (Höchst Ceram Tec, Wunsiedel, Germany) with a diameter of 6 mm were used as antagonists. The contact point of the steatite ball on the Michigan splint was adjusted to the central area of tooth 15 (Figure 5).

**Figure 6:**
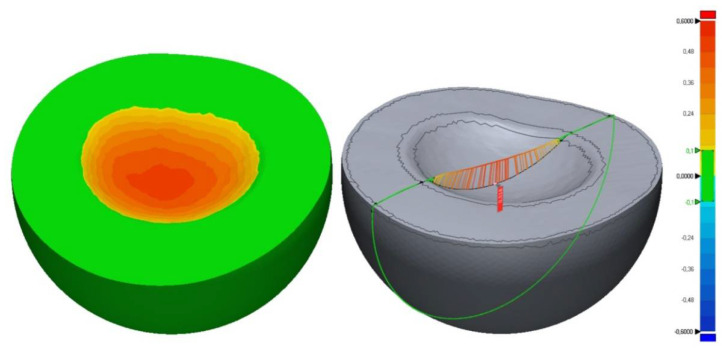
Exemplary scan of the 3D topography at the tooth region 15. (green: 0–100 µm, yellow: 100–240 µm, orange: 240–480 µm, and red: 480–600 µm).

After the artificial loading, a second scan of the area of tooth 15 was performed and again STL files were generated from the samples. The wear occurring during the artificial loading was calculated with a 3D evaluation software (Geomagic Control X 2020.0, Morrisville, NC, USA). For this, the datasets generated before and after the artificial loading were superimposed using the software’s best fit alignment. The maximum vertical wear [µm] was calculated and topographical profiles were created to illustrate the wear (Figure 6).

### 2.5. Statistical Analyses

Descriptive statistical analyses (median, mean ± standard deviation) were calculated for the duration needed to fabrication the splints (dentist and dental technician), the overall fit, the static occlusion and the wear. Paired t-tests were used to compare the duration (dentist, dental technician) for the conventional and digital workflows as well as for the wear of the two different materials. For statistical analyses of the overall fit and static occlusion, the sign test was used. All analyses were performed with a statistical evaluation software (STATA 16.1, StataCorpLLC, College Station, TX, USA). The level of statistical significance was set to *p* < 0.05.

## 3. Results

### 3.1. Duration for the Splint Fabrication

**Figure 7:**
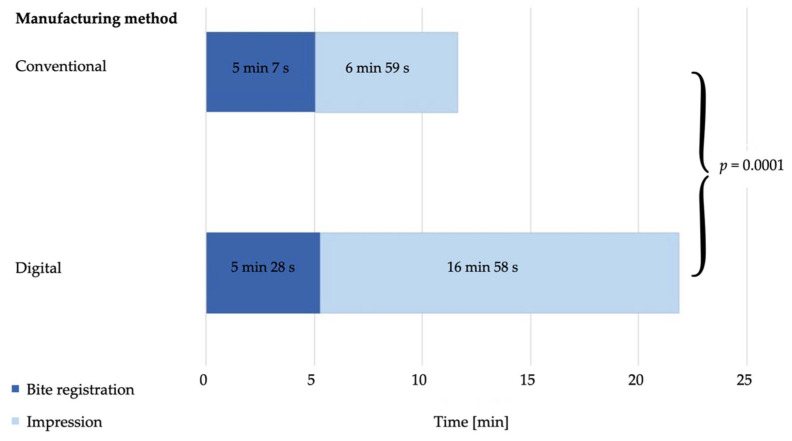
Mean time requirement (dentist) for the bite registration and impressions in minutes (min) and seconds (s).

**Figure 8:**
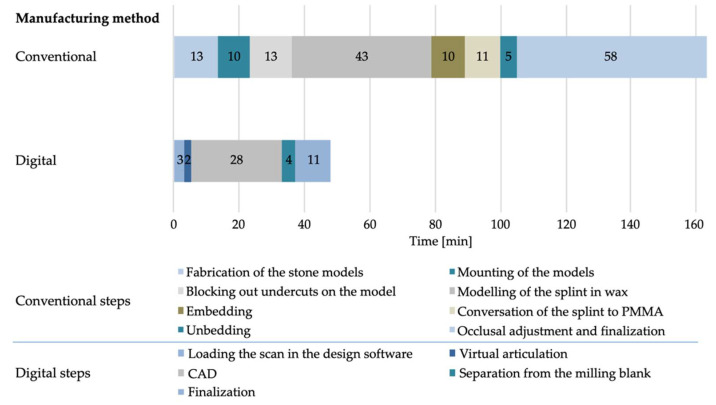
Time requirement for the dental technician for the different steps in minutes (min).

The total time required by the dentist for the impressions and bite registrations differed significantly (*p* = 0.0001) between the two workflows. The dentist needed on average 12 min 6 s ± 2 min 27 s for the conventional workflow and 22 min 26 s ± 3 min 19 s for the digital workflow (Figure 7). There was a statistically significant time difference for the bite registration between the conventional (5 min 7 s ± 39 s) and the digital workflow (5 min 28 s ± 39 s; *p* = 0.01). Likewise, a significant difference was found for the impression times (conventional: 6 min 59 s ± 1 min 28 s, digital: 16 min 58 s ± 2 min 54 s; *p* = 0.0001). The time required by the dental technician for the fabrication of the splints also differed significantly (*p* = 0.0001) between the two different workflows (Figure 8). The conventional production process took on average 163 min 32 s ± 17 min 24 s, while the digital production was 47 min 52 s ± 8 min 46 s (Table 2).

### 3.2. Fit of the Splints

One of the conventionally manufactured Michigan splints showed a perfect fit (score 1), seven had a good fit (score 2), three had a poor fit (score 3), and four could not be inserted (score 4). In contrast, 14 Michigan splints fabricated using the digital workflow had a perfect fit (score 1) and one Michigan splint had a poor fit (score 3). The overall fit between the conventionally (median score: 2, mean score: 2.6 ± 0.9) and digitally (median score: 1, mean score: 1.1 ± 0.5) fabricated splints was significantly different (*p* = 0.002) (Table 2).

Two conventionally-fabricated Michigan splints had a perfect static occlusion (1), seven had a good static occlusion (2), and two had a poor static occlusion (3). In four conventionally-fabricated Michigan splints, the static occlusion could not be evaluated due to a poor general fit (4). Six Michigan splints of the digital workflow demonstrated a perfect static occlusion (1), seven had a good static occlusion (2), and two had a poor static occlusion (3). The results of the static occlusion between the conventionally (median score: 2, mean score: 2.5 ± 1.0) and digitally (median score: 2, mean score: 1.7 ± 0.7) fabricated splints, however, did not differ significantly (*p* = 0.11) (Table 2).

### 3.3. Occlusal Wear

The average wear was 518 µm ± 4 µm for the material of the conventionally manufactured splints and 539 µm ± 5 µm for the material of the digitally-fabricated splints (*p* = 0.26) (Table 2).

## 4. Discussion

Studies investigating the effectiveness of digitally-fabricated Michigan splints confirmed similar treatment success compared to conventionally-fabricated Michigan splints [27,29,37]. However, differences in conventional and digital fabrication workflows exist regarding the duration for the fabrication of the splints. According to the knowledge of the authors, the present investigation comparing a conventional to a digital workflow seems to be the first investigating the production time of the splints, the overall fit, the static occlusion, and the wear after 1.2 million loading cycles in an artificial chewing simulator.

Since this study was performed as an in vitro investigation, a standardized setting (dental simulation unit) and laboratory production process was established allowing a comparison of the two different manufacturing methods. Many clinical influences such as saliva, restricted space availability, missing teeth, shiny restorations, anatomic limitations, jaw movement, and mobile soft tissue influencing the impression results could be eliminated [38,39]. However, the exclusion of those clinical influences which mainly concern the dentist’s work, should be considered when interpreting the result in a clinical context and transferring them into an in vivo scenario. Nevertheless, for the manufacturing of the splints itself, it is irrelevant whether the impressions were made in an in vivo or in vitro setting since the technicians working steps are based on stone cast or virtual model.

In the present investigation, the digital workflow required significantly less time for the splint fabrication than the conventional one, although the time required for the computerized optical impression was significantly more than for the conventional impressions. In this context, Dedem and Türp showed that digitally manufactured Michigan splints can be produced time- and cost-efficiently suggesting that the digital workflow is the favorable production method in the future [17].

The production of Michigan splints comprises several clinical and laboratory steps. The most crucial step is the impression making of the jaws. Among others, Patzelt et al. [40] compared in an in vitro study the duration for conventional and computerized optical impressions of full-arches. The authors found that computerized optical impressions are less time consuming than conventional impressions (digital: 17–20 min, conventional: 21–30 min). Likewise, in an in vitro study evaluating a single-implant impression, Joda et al. [41] reported that computerized optical impressions are less time-consuming than conventional impressions (digital: 4 min 53 s, conventional: 10 min 9 s). Furthermore, Mangano et al. reported about the time efficiency of conventional and computerized optical impressions [42]. The authors revealed no time difference between conventional and computerized optical impressions (3–5 min). In contrast, the present study observed that less time was required for the conventional impressions. However, the difference to the computerized optical impressions was only 20 s on average. One of the reasons for this difference is obviously caused by the kind of the conventional impression material used. Patzelt et al. and Joda et al. used precision impression materials (polyether, silicone), whereas alginate was used in the present investigation [40,41]. Precision impression materials have per se a longer processing time than alginate materials. However, in the present study, the time required for the computerized optical impressions of the upper and lower full-arches (~17 min) was similar to the full-arch optical impressions in the investigation of Patzelt et al. (17–21 min) [40]. Another reason for the increased time required for the computerized optical impressions might be the handling of advanced technologies by clinicians and the associated learning curve [41,42]. Since in the present study the computerized optical impressions were taken by a clinician experienced in intraoral optical impression making, no learning curve could be observed and there was no difference in the time between the first and last optical impressions.

For computerized optical impressions, steps like impression disinfection, packaging, and physical transfer to a laboratory can be skipped saving additional time [40,41,43].

In contrast to the increased time requirement for the dentist in the digital workflow, the working time for the dental technician was decreased by 71% due to the reduced number of working steps during the digital manufacturing process [17,27,28]. A case report by Venezia et al. confirmed these results of time reduction when applying digital manufacturing processes [28].

Another major advantage of the digital workflow compared to the conventional workflow was the significantly better fit of the splints in the present investigation. Although some previous in vitro and in vivo studies have shown comparable results between conventionally and digitally produced splints, they have not classified them with a score system [27,29,44]. In those studies, the splints were solely tested for clinical acceptance. A study by Wesemann et al. [44] examining conventionally and digitally manufactured splints (subtractive and additive) concluded that all three manufacturing methods showed comparable results regarding the fit. However, the authors showed a superiority of subtractive manufactured splints to additive manufactured splints in regard to static occlusion points [44].

Based on the investigation of Wesemann et al. [44], a subtractive CAM process was chosen in the present investigation. Industrially manufactured PMMA blanks feature a higher material density and an increased polymerization grade. Inhomogeneities and pores were rarely found in Michigan splints produced from industrial processed blanks compared to the ones fabricated using a conventional workflow such as injection molding [45,46]. Considering environmental issues, it has to be considered that only two Michigan splints can be milled out of one blank. This leads to a non-recyclable material waste of approximately 70% [30]. This definitely represents an environmental downside of this fabrication method, for this future research should focus on additive manufacturing processes [22].

Considering the materials’ wear, the present study was limited to a comparison of subtractive and conventionally manufactured splints. However, there was no significant difference in the wear behavior between the two materials after 1.2 million loading cycles. In previous studies [30,31,32], the wear of different splint materials (conventional, subtractive, or additive) was compared in computer-controlled chewing simulators. Yet, the study designs and parameters (especially load and number of cycles) were different. In the present study, an occlusal load of 50 N was chosen. This value is considered the standard value when using artificial chewing simulators [30,47,48]. Beyond, Huettig et al. used merely 5 N in their investigation [32]. After 5000 chewing cycles, the study concluded that the wear of the conventionally manufactured splint material (111.4 µm) and the subtractive manufactured splint material (85.7 µm) differed significantly. Lutz et al. investigated the wear and failure load of printed, milled, and conventional splint materials [30]. The investigation was carried out with the same load as in the present investigation (50 N), though, with cycle numbers of 20,000 and 120,000. They revealed that the material loss was higher of for the printed material (2.8 mm^3^) was higher compared to the conventional (1.2 mm^3^), and milled materials (1.8 mm^3^) after 120,000 cycles. A study by Wesemann et al. [31] that investigated the wear of conventionally manufactured, milled, and printed splint materials after 200,000 cycles also showed no significant difference in wear between the three materials ranging between 550 µm (conventional) and 590 µm (milled).

The results of the present in vitro investigation suggest a superiority of the digital manufacturing workflow for Michigan splints in terms of overall time efficiency, fit, and static occlusion. Although the use of dental simulation units allows a certain degree of comparability to a clinical setting, the results need to be confirmed in vivo to appreciate the influences of a clinical setting

## 5. Conclusions

Within the limitations of the present in vitro study, it can be concluded that a digital workflow in toto is more time efficient for the fabrication of occlusal splints than a conventional workflow. The fit of the digitally-fabricated splints was superior to the conventionally-fabricated ones. The wear of the two different materials showed comparable results.

## Figures and Tables

**Table 1:** Score system for the overall fit and static occlusion.

Score	Overall Fit	Static Occlusion
1	No Further Adaption Required	9–10 Contact Points
2	Subtractive Adaption Possible	7–8 Contact Points
3	Additive Rework Necessary	5–6 Contact Points
4	New Fabrication Necessary	≤4 Contact Points

**Table 2:** Results for the conventional and digital workflow showing the duration for the dentist and the dental technician, the overall fit, the static occlusion, and the wear.

	N		Median		Mean		SD	
	c	d	c	d	c	d	c	d
Time Bite Registration	15	15	4 min 53 s	5 min 40 s	5 min 7 s	5 min 28 s	39 s	39 s
Time Impression	15	15	6 min 17 s	17 min 45 s	6 min 59 s	16 min 58 s	1 min 28 s	2 min 54 s
Time Dentist–Total	15	15	11 min 10 s	23 min 25 s	12 min 6 s	22 min 26 s	2 min 27 s	3 min 19 s
Time Dental Technician	15	15	155 min 00 s	47 min 4 s	163 min 32 s	47 min 52 s	17 min 24 s	8 min 46 s
Overall Fit	15	15	2	1	2.6	1.1	0.9	0.5
Static Occlusion	15	15	2	2	2.5	1.7	1.0	0.7
Wear	8	8	517 µm	559 µm	518 µm	539 µm	4 µm	5 µm

N = number of splints, c = conventional workflow, d = digital workflow, and SD = standard deviation.

## Data Availability

Not applicable.

## References

[1] Okeson J. (2004). Bell’s Orofacial Pains: The Clinical Management of Orofacial Pain.

[2] Carlsson G.E. (1985). Long-term effects of treatment of craniomandibular disorders. Cranio.

[3] Ash M.M. (2006). Klassifikation, Epidemiologie, Ätiologie, Symptome, Diagnose und Pathophysiologie von TMD und CMD. Schienentherapie: Evidenzbasierte Diagnostik und Behandlung bei TMD und CMD.

[4] Alencar F., Becker A. (2009). Evaluation of different occlusal splints and counselling in the management of myofascial pain dysfunction. J. Oral Rehabil..

[5] Bumann A., Lotzmann U., Rateitschak K.-H., Wolf H.F. (2016). Funktionsdiagnostik und Therapieprinzipen. Farbatlanten der Zahnmedizin, Stuttgart.

[6] Fricton J., Look J.O., Wright E., Alencar F.G.P., Chen H., Lang M., Ouyang W., Velly A.M. (2010). Systematic review and meta-analysis of randomized controlled trials evaluating intraoral orthopedic appliances for temporomandibular disorders. J. Orofac. Pain.

[7] Kuzmanovic Pficer J., Dodic S., Lazic V., Trajkovic G., Milic N., Milicic B. (2017). Occlusal stabilization splint for patients with temporomandibular disorders: Meta-analysis of short and long term effects. PLoS ONE.

[8] Anderson G.C., Schulte J.K., Goodkind R.J. (1985). Comparative study of two treatment methods for internal derangement of the temporomandibular joint. J Prosthet. Dent..

[9] Wassell R.W., Adams N., Kelly P.J. (2006). The treatment of temporomandibular disorders with stabilizing splints in general dental practice: One-year follow-up. J. Am. Dent. Assoc..

[10] Freesmeyer W.B. (2004). Okklusionsschienen. Zahnärztl. Mitt..

[11] Ordelheide A., Bernhardt O. (2009). The effectiveness of occlusal splints for the treatment of craniomandibular dysfunctions—An overview of national and international publications. J. Craniomandib. Funct..

[12] Schindler H.J., Svensson P., Türp J.C., Sommer C., Hugger A. (2007). Myofascial Temporomandibular Disorder Pain Pathophysiology and Management. The Puzzle of Orofacial Pain: Integrating Research into Clinical Management.

[13] Schindler H.J., Hugger A., Kordaß B., Türp J.C. (2014). Splint therapy for temporomandibular disorders: Basic principles. J. CranioMandib. Funct..

[14] Schindler H.J., Türp J.C., Nilges P., Hugger A. (2013). Therapie bei Schmerzen der Kaumuskulatur: Aktualisierung der Empfehlungen. Schmerz.

[15] Ash M.M., Ramfjord S.P. (1998). Reflections on the Michigan splint and other intraocclusal devices. J. Mich. Dent. Assoc..

[16] Geering A.H., Lang N.P. (1978). Die Michigan-Schiene, ein diagnostisch und therapeutisches Hilfsmittel bei Funktionsstörungen im Kausystem: Herstellung im Artikulator und Eingliederung am Patienten. Schweiz. Mon. Zahnmed..

[17] Dedem P., Türp J.C. (2016). Digital Michigan splint—From intraoral scanning to plasterless manufacturing. Int. J. Comput. Dent..

[18] Ender A., Mehl A. (2011). Full arch scans: Conventional versus digital impression-an in-vitro study. Int. J. Comput. Dent..

[19] Güth J.-F., Edelhoff D., Schweiger J., Keul C. (2016). A new method for the evaluation of the accuracy of full-arch digital impressions in vitro. Clin. Oral Investig..

[20] Patzelt S.B.M., Emmanouilidi A., Stampf S., Strub J.R., Att W. (2014). Accuracy of full-arch scans using intraoral scanners. Clin. Oral Investig..

[21] Zimmermann M., Koller C., Rumetsch M., Ender A., Mehl A. (2017). Precision of guided scanning procedures for full-arch digital impressions in vivo. J. Orofac. Orthop..

[22] Methani M.M., Cesar P.F., de Paula Miranda R.B., Morimoto S., Özcan M., Revilla-León M. (2020). Additive manufacturing in dentistry: Current technologies, clinical applications, and limitations. Curr. Oral Health Rep..

[23] Joda T., Brägger U. (2016). Patient-centered outcomes comparing digital and conventional implant impression procedures: A randomized crossover trial. Clin. Oral Implant. Res..

[24] Beuer F., Schweiger J., Neumeier P., Güth J.-F., Edelhoff D. (2012). Digitales Update: Wo stehen die Intraoralscanner heute?. ZWR.

[25] Burhardt L., Livas C., Kerdijk W., van der Meer W.J., Ren Y. (2016). Treatment comfort, time perception, and preference for conventional and digital impression techniques: A comparative study in young patients. Am. J. Orthod. Dentofac. Orthop..

[26] Grünheid T., McCarthy S.D., Larson B.E. (2014). Clinical use of a direct chairside oral scanner: An assessment of accuracy, time, and patient acceptance. Am. J. Orthod. Dentofac. Orthop..

[27] Berntsen C., Kleven M., Heian M., Hjortsjö C. (2018). Clinical comparison of conventional and additive manufactured stabilization splints. Acta Biomater..

[28] Venezia P., Lo Muzio L., de Furia C., Torsello F. (2019). Digital manufacturing of occlusal splint: From intraoral scanning to 3D printing. J. Osseointegr..

[29] Pho Duc J.M., Hüning S.V., Grossi M.L. (2016). Parallel randomized controlled clinical trial in patients with temporomandibular disorders treated with a CAD/CAM V versus a conventional S stabilization splint. Int. J. Prosthodont..

[30] Lutz A.-M., Hampe R., Roos M., Lümkemann N., Eichberger M., Stawarczyk B. (2019). Fracture resistance and 2-body wear of 3-dimensional-printed occlusal devices. J. Prosthet. Dent..

[31] Wesemann C., Spies B.C., Sterzenbach G., Beuer F., Kohal R., Wemken G., Krügel M., Pieralli S. (2020). Polymers for conventional, subtractive, and additive manufacturing of occlusal devices differ in hardness and flexural properties but not in wear resistance. Dent. Mater..

[32] Huettig F., Kustermann A., Kuscu E., Geis-Gerstorfer J., Spintzyk S. (2017). Polishability and wear resistance of splint material for oral appliances produced with conventional, subtractive, and additive manufacturing. J. Mech. Behav. Biomed. Mater..

[33] Hellmann D., Türp J.C., Schindler H.J., Türp J.C. (2017). Praxis der Schienenherstellung. Konzept Okklusionsschiene: Basistherapie bei Schmerzhaften Kraniomandibulären Dysfunktionen.

[34] Türp J.C. (2011). Step by Step Kieferrelationsbestimmung. Quintessenz Zahntech..

[35] Türp J.C. (2002). Okklusionsschienen. Dtsch. Zahnärztl. Z..

[36] Broetzner H.-P. (2021). Sirona Connect—Training: Digitale Abformung. https://assets.dentsplysirona.com/websites/sirona-connect/pdf/sirona-connect-training_DE.pdf.

[37] Salmi M., Paloheimo K.-S., Tuomi J., Ingman T., Mäkitie A. (2013). A digital process for additive manufacturing of occlusal splints: A clinical pilot study. J. R. Soc. Interface.

[38] Ender A., Attin T., Mehl A. (2016). In vivo precision of conventional and digital methods of obtaining complete-arch dental impressions. J. Prosthet. Dent..

[39] Kernen F., Schlager S., Seidel Alvarez V., Mehrhof J., Vach K., Kohal R., Nelson K., Flügge T. (2021). Accuracy of intraoral scans: An in vivo study of different scanning devices. J. Prosthet. Dent..

[40] Patzelt S.B.M., Lamprinos C., Stampf S., Att W. (2014). The time efficiency of intraoral scanners: An in vitro comparative study. J. Am. Dent. Assoc..

[41] Joda T., Lenherr P., Dedem P., Kovaltschuk I., Brägger U., Zitzmann N.U. (2017). Time efficiency, difficulty, and operator’s preference comparing digital and conventional implant impressions: A randomized controlled trial. Clin. Oral Implant. Res..

[42] Mangano F., Gandolfi A., Luongo G., Logozzo S. (2017). Intraoral scanners in dentistry: A review of the current literature. BMC Oral Health.

[43] Burzynski J.A., Firestone A.R., Beck F.M., Field H.W., Deguchi T. (2018). Comparison of digital intraoral scanners and alginate impressions: Time and patient satisfaction. Am. J. Orthod. Dentofac. Orthop..

[44] Wesemann C., Spies B.C., Schaefer D., Adali U., Beuer F., Pieralli S. (2021). Accuracy and its impact on fit of injection molded, milled and additively manufactured occlusal splints. J. Mech. Behav. Biomed. Mater..

[45] De Souza Leão R., de Moraes S.L.D., da Silva Aquino K.A., Isolan C.P., Casado B.G.S., Montes M.A.J.R. (2018). Effect of pressure, post-pressing time, and polymerization cycle on the degree of conversion of thermoactivated acrylic resin. Int. J. Dent..

[46] Nguyen J.-F., Migonney V., Ruse N.D., Sadoun M. (2012). Resin composite blocks via high-pressure high-temperature polymerization. Dent. Mater..

[47] Lambrechts P., Debels E., van Landuyt K., Peumans M., van Meerbeek B. (2006). How to simulate wear? Overview of existing methods. Dent. Mater..

[48] Wimmer T., Huffmann A.M.S., Eichberger M., Schmidlin P.R., Stawarczyk B. (2016). Two-body wear rate of PEEK, CAD/CAM resin composite and PMMA: Effect of specimen geometries, antagonist materials and test set-up configuration. Dent. Mater..

